# A Solace for Solis

**Published:** 1892-09

**Authors:** 


### A SOLACE FOR SOLIS.

The question of advertising in medical journals is being discussed somewhat in the medical societies, and there appears to be a great diversity of opinion on the subject. As expressive of two extremes of sentiment, and as being of interest to our readers, as well as very interesting reading, we reproduce from our esteemed contemporary, the *Medical Mirror*, Dr. Love's sizing up of Dr. Solis Cohen's famous resolution forninst the subject in general, and the *Journal of the American Medical Association* in particular, and the resolution itself. Dr. Love is a graceful writer, and when he has a suitable subject in hand, he is deft at the art of flaying. In this instance, he makes the fur fly, and peels off the cuticle with the hand of an expert:

"A modern Solomon of Philadelphia, who is the son of his father, and that is saying much, has elected himself to be the Moses to lead the State profession of Pennsylvania away from the blandishments of the medical purveying guild.

"In a flaming blast, suggestive of Mount Sinai, this modern Moses winds up as follows:

"Far more iniquitous and far more dangerous to society is the wily manufacturer that advertises 'to the profession only.' Whether he ostentatiously holds secret the composition of his nostrum, or whether with pretended frankness he describes it with an appellation that means nothing, or publishes a formula that cannot be carried out, his object is the same; he seeks to make the physician's the hand whereby he may reach pockets shut from the coarser methods of the Warners, the Pinkhams, and the Jaynes; for, after all, it is the minority that can be deluded by the flaring posters of 'Wizzard Oil,' or the lying testimonials of 'Tonic Vermifuge.' When a sick man applies to a physician, thinking that thereby he will secure the benefit of special knowledge brought to bear upon the conditions of the individual case, intrusting to the conscience of his medical adviser his health and his life, he is entitled to the skill and the thought for which he pays, and that he deems himself to be receiving. He certainly deserves better treatment than to be handed over to the mercies of 'antikamnia,' or 'febricide,' or quickine,' or 'gleditschine,' or 'Freligh's tablets,' or 'listerine,' or any other of the unholy crew. If such is to be his fate, let him have the satisfaction of buying the worthless or poisonous stuff direct, without the sham of a professional consultation, and without paying a purchaser's commission to the medical sales-agent.

"He then announces that at the coming meeting of the Pennsylvania State Medical Society, he will offer the following resolutions:

"*Resolved*, That the Medical Society of the State of Pennsylvania hereby expresses its highest disapprobation of the practice of giving certificates or testimonials to secret preparations alleged to be of medicinal virtue, and calls the attention of the affiliated county societies to the fact that such action on the part of members of the said societies is in derogation of the dignity of the profession, and in violation of the letter and the spirit of the Code of Ethics of the American Medical Association and of this Society.

"*Resolved*, That this Society likewise expresses its disapprobation of the practice of inserting advertisements of secret preparations in the columns of medical journals, such action being an insult to the intelligence of the profession, and a degradation of journals indulging therein to the level of the patent medicine almanac. Especially to be condemned is the action of the *Journal of the American Medical Association* in admitting such advertisements.

"*Resolved*, That copies of these resolutions, duly attested by the permanent secretary, be sent to all county societies in affiliation with this Society, to the American Medical Association, to State Medical Societies in affiliation therewith, and to the publishers and editors of American medical journals.

"Let the medical profession, composed as it is of a body of infants, get down upon its marrow bones and thank the Almighty that there are in the profession a few men of ability capable of perceiving the dangers which they have overlooked; ready to give them 'Solis' for their long suffering at the hands of the wily medical purveyors. By all means, let the amendments to the Code of Ethics be adopted. Yes, and as many more amendments as may be evolved. Verily, verily, it may be said unto the medical profession, 'Oh, Lord! Oh, Lord! How long is the profession to be burdened by those who escape the fool killer.'

"While the bulk of the profession is feeling more and more every day that the best code is the one that can be expressed in the fewest clauses, and that the individual or community is best governed that is governed the least, and that the unwritten law that governs gentlemen is all that is necessary for any educated gentleman in any calling.

"There is ever and anon coming to the front some self-elected shouter for more legislation. All the rules and regulations in Christendom will not affect a man in his employment of the tools necessary for his work. If he finds a thing good in the treatment of disease, he will be apt to use it, no matter who makes it; no matter what the combination of remedies may be; no matter who gets the benefits of its sale.

"These various outbursts of the Solomons and other representatives of wisdom and plu-perfection in the profession would be amusing were it not annoying to the sensible men of our guild. We fancy we can hear these mighty Solons say to their fellows immediately near them: 'Do not think too highly of our supreme goodness; do not admire our lofty attitudes too much; do not adore our shape and talents too completely; we deserve no credit for it. The honor all belongs to God who made us so.'"

In our October number, we will have something to say on advertising, from our own standpoint as a medical publisher.

